# Overexpression of piRNA Pathway Genes in Epithelial Ovarian Cancer

**DOI:** 10.1371/journal.pone.0099687

**Published:** 2014-06-16

**Authors:** Shu Ly Lim, Carmela Ricciardelli, Martin K. Oehler, Izza M. D. De Arao Tan, Darryl Russell, Frank Grützner

**Affiliations:** 1 Robinson Research Institute, School of Molecular and Biomedical Sciences, University of Adelaide, Adelaide, SA, Australia; 2 Discipline of Obstetrics and Gynaecology, Robinson Research Institute, University of Adelaide, Adelaide, SA, Australia; University of Alabama at Birmingham, United States of America

## Abstract

The importance of the Piwi-interacting RNA (piRNA) pathway for germ cell maintenance, genome integrity, DNA methylation and retrotransposon control raises possible roles of this pathway in cancer. Indeed aberrant expression of human PIWI orthologs and *Maelstrom* has been observed in various cancers. In this study we explored the expression and function of piRNA pathway genes in human ovarian cancer, based on our recent work, which showed widespread expression of piRNA pathway genes in the mammalian. Our work shows that *PIWIL1* and *MAEL* expression is significantly increased in malignant EOC (n = 25) compared to benign tumor tissues (n = 19) and normal ovarian tissue (n = 8). The expression of *PIWIL3* is lower in malignant and benign tissues when compared to normal ovary. Sequencing of *PIWIL1* transcript revealed that in many tumors deletion of exon 17 leads to the introduction of a premature stop codon in the PIWI domain, likely due to a splicing error. *In situ* hybridization on tumor sections revealed that *L1*, *PIWIL1*, *2* and *MAEL* are specifically expressed in epithelial cells (cancerous cells) of EOC. Furthermore, *PIWIL2* and *MAEL* are co-expressed in the stromal cells adjacent to tumor cells. Since *PIWIL1* and *MAEL* are up regulated in malignant EOC and expressed in the epithelial cells, we investigated if these two genes affect invasiveness of ovarian cancer cell lines that do not normally express these genes. *PIWIL1* and *MAEL* were transiently over expressed in the ovarian cancer cell line SKOV3, followed by real-time measurements of cell invasiveness. Surprisingly both *PIWIL1* and *MAEL* over expression decreased the invasiveness of SKOV3 cells. Our findings support a growing body of evidence that shows that genes in this pathway are upregulated in cancer. In ovarian cancer we show for the first time that *Piwil1* transcript may often be abnormal result in non functional product. In contrast to what has been observed in other cell types, we found that *PIWIL1* and *MAEL* have a repressive effect on cell invasiveness.

## Introduction

Ovarian cancer is the most lethal gynaecological cancer, and the fifth leading cause of cancer-related death among women in the Western World [1]. The five-year relative survival rate for women with ovarian cancer is only around 40% [1]. Ovarian cancers are heterogeneous tumors which exhibit distinct morphological characteristics, genetic mutations and origins. There are three major types of ovarian cancer - epithelial, germ cell and sex cord stromal tumors. Ovarian germ cell tumors and sex cord stromal tumors comprise 10% of ovarian cancers, and are derived from primitive ovarian germ cells or mesenchymal cells in the sex-cord derived tissue of the ovary, respectively [2]. EOCs account for more than 90% of ovarian malignancies. Based on histology EOCs are classified into four main subtypes (serous, mucinous, endometroid and clear cell carcinomas) with over 70% of the cases diagnosed as serous carcinomas (SCs) [3].

Alterations of the epigenetic landscape such as global DNA hypomethylation and gene specific DNA hypermethylation are frequently reported in cancer cells [4]. Global DNA hypomethylation largely affects the intergenic and intronic regions of the genome, especially repeat sequences and transposable elements (TEs), which account for about 55% of the human genome [5], including 17% *L1* repeats [6]. In somatic cells, DNA methylation of TEs is crucial to prevent their expression which can jeopardise integrity of the genome. During germ cell development, there is a transient period of global DNA hypomethylation which results in temporary activation of TEs [7].

The piRNA pathway is evolutionarily highly conserved in metazoa and consists of 21–26 nt piRNAs which bind PIWI proteins to mediate posttranslational control of TE expression and play a role in epigenetic changes (such as DNA methylation) via interaction with other proteins (such as MAEL or HP1) [8], [9]. In mammals there are several *Piwi like* (*Piwil*) genes (three in mice, four in humans). Expression of *Piwil* genes and piRNA pathway associated genes has been demonstrated at various stages of germ cell development in particular in testis. *Piwil1* (*Miwi*) has previously been identified as a gene exclusively expressed in mouse testis and essential for spermatogenesis [10]. More recently expression of *Piwil1* has also been demonstrated in mouse and human ovary [11]. Mutation of *Piwil2*, *Piwil4* and *Mael* in mouse leads to similar phenotypes including elimination of TE DNA methylation and male sterility [8]. There is now growing evidence of piRNA activity also in the mammalian ovary, however knock out experiments of piRNA pathway gene in mice so far has only led to male sterility [12]–[14].

There is mounting evidence of piRNA pathway activity in various cancers. Expression of *PIWIL1* and *PIWIL2* has been found in a wide range of human cancers such as stomach, breast, gastrointestinal tract and endometrium [15]–[18] and recently also in ovarian carcinoma [19]. Increased expression of *PIWIL1* is associated with enhanced tumor growth [20] increased tumor grades [21], poor diagnostic outcomes [22] and mortality [23]. *PIWIL2* is widely expressed in early-stage breast and cervical cancers and pre-cancerous stem cells involved in tumorigenesis [18]. The expression pattern of *PIWIL3* and *4* has only been studied in colon cancers [24]. *Maelstrom* (*MAEL*) is a known testis cancer gene [25].

Our finding of expression in ovarian somatic cells [11] together with decreased DNA methylation at *L1* promoter region associated with the progression of cervical and uterus cancers [26] and poor prognosis in ovarian carcinomas [27] led us to investigate whether piRNA pathway genes could play a role in EOCs. In this study, we investigated the expression of *PIWIL1*–*4* and *MAEL* in 25 EOC, 19 benign tumor samples, and 8 normal ovarian tissues. We found significantly elevated expression of *PIWIL1* and *MAEL* in EOC compared to benign and normal ovarian tissues. However, *PIWIL1* transcripts in EOC may not be functional due to deletions identified in the PIWI domain of this transcript. Furthermore, overexpression of *PIWIL1* and *MAEL* suggests a role of these genes in reducing ovarian cancer cells invasiveness *in vitro*.

## Materials and Methods

### Tissues

Total RNAs from human testis and pre-menopause ovarian tissues were purchased from Stratagene (USA). Malignant, benign and normal ovarian tissues (Table 1 and Table S3) were collected with informed written consent of the patient and approval by the Ethics Committee of the Royal Adelaide Hospital, Adelaide, South Australia.

**Table 1:** Age and cancer stage of EOC, benign tumors and normal ovaries.

Tissue	n	Median of patient age in years (range)	Cancer stage
Serous carcinomas	25	60 (37–75)	See Table S3
Benign ovarian lesions	19	60 (36–88)	-
Normal ovaries	8	48 (44–76)	-

### Cell lines

Human ovarian cancer cell lines SKOV3 and OVCAR3 were purchased from American Type Culture Collection (ATCC, USA) while OVCAR5 was obtained from Dr. Thomas Hamilton (Fox Chase Cancer Center, Philadelphia, PA) Johnson et al Cancer Res 57: 850–856 1997. Cell lines were maintained in RPMI 1640 medium supplemented with L-glutamine (2 mM), Penicillin-streptomycin (100 U/ml, 100µg/ml) (Sigma). SKOV3 and OVCAR3 cells were supplemented with 5% FBS (Invitrogen) whilst OVCAR5 cells were supplemented with 10% FBS and 7.5 µg/ml insulin. All cell lines were maintained at 37°C in a humid chamber with 5% CO_2_.

### RNA isolation and cDNA synthesis

RNA was isolated from tissues and cell lines using TRIzol reagent (Invitrogen) following the manufacturer's instructions. RNA was resuspended in nuclease free water, and stored at -80°C. RNA was treated with DNase I (New England Biolabs) before reverse transcription. 1µg of RNA was used to obtain cDNA using the Super Script III First-strand Synthesis System (Invitrogen) following the manufacturer’s protocol. Briefly, RNA was incubated with 1µl of 50 µM oligo(dT)_20_ and 1µl of 10 mM dNTPs for 10 mins at 65°C. After incubation, 4µl of 5x RT buffer, 2µl of dithiothreitol (DTT, 0.1 M), 1µl of RNaseOUT (40 U/μl) and 1µl of Super Script III RT enzyme (200 U/μl) were added and incubated for 50 mins at 50°C. The reaction was terminated at 85°C for 5 mins. cDNAs were stored at −20°C.

### Gene expression analyses

RT-PCR was performed to determine the level of mRNA for the five piRNA pathway genes (Table S1) and beta actin (*ACTB*) in 52 patient samples and 3 ovarian cancer cell lines. Each 25µl reaction contained 200 ng of cDNA, 5µl of 5x Go Taq Green Master Mix (Promega), 1µl of 5 mM dNTP solution (Roche), 0.5µl of each primer (20 pmol/μl) and 0.5µl Taq DNA polymerase. The PCR conditions were the same for all genes, except the annealing temperature (Table S1), and were as follows: initial denaturation at 95°C for 2 mins, 95°C for 30 secs, annealing at gene specific temperature for 30 secs (Table S1), extension at 72 °C for 1 min, 32 cycles. PCR of *ACTB* control was performed with 27 cycles, followed by 5 mins of final extension at 72 °C. The PCR products were visualized on a 1.5% agarose gel stained with ethidium bromide. Band intensity was measured (Quantity One program, Version 4, Bio-Rad), and the relative intensity of each gene was normalized to that of *ACTB*. PCR products were confirmed by sequencing (Big Dye Terminator v3.1 cycle sequencing kit, Applied Biosystems). RT–PCR was performed in triplicate for each primer pair.

### Statistical analyses of gene expression

Data are presented as median relative expression (first quartile to third quartile). The transcript level of each gene was normalized against *ACTB*. Kolmogorov-Smirnov and Shapiro-Wilk tests suggested that the expression level of each gene from all of the 52 samples is not normally distributed. Thus, a Kruskal-Wallis test, which uses a non-parametric method, was used to compare the gene expression from malignant, benign and normal groups. The Spearman's correlation test was performed to investigate the correlation between a patient's age and consequent gene expression. R-value closer to 1 indicates a stronger positive correlation, whereas an R-value closer to -1 shows a stronger negative correlation. In the Kruskal-Wallis and Spearman's tests, a P-value of <0.05 was considered statistically significant.

### RNA *in situ* hybridization

Probes were labelled with digoxigenin-11-UTP (Roche Diagnostics) according to the manufacturer's protocol. Formalin-fixed paraffin-embedded tissue sections were deparaffinised in xylene and subsequently washed in graded ethanol. All solutions were prepared in diethylpyrocarbonate (DEPC)-treated H_2_O to inactivate RNase activity. mRNA-bound nucleoproteins were removed by proteinase K incubation (1.2µg/ml) (Roche Diagnostics) for 30 mins at 37 °C. Slides were washed with 1x PBS and acetylated (1 M triethanolamin/0.178% HCl/0.25% acetic anhydride). Slides were prehybridised at 65°C for 2 hrs with prehybridization buffer (50% deionized formamide/2x SSC/1x Denhardt's solution/0.005 M phosphate buffer/10% dextran sulphate/1 mg/ml yeast total RNA/1 mg/ml salmon sperm DNA) and hybridised with about 200 ng of cRNA probe in prehybridization buffer overnight at 50°C in a humid chamber. Slides were washed in 2x SSC, 1x SSC, 0.5x SSC and 0.1x SSC for 15 mins each at 50°C. To remove unbound cRNA probes, the tissue was subjected to RNase A (150µg/ml) digestion for 30 mins at 37°C. Specifically bound cRNA probes were detected using an anti-DIG antibody coupled to alkaline phosphatase with nitrobluetetrazolium chloride/X-phosphate-5-bromo-4-chloro-3-indolylphosphate (NBT/BCIP) (Roche Diagnostics) as chromogenic substrate.

### Cloning and construct preparation for invasion analyses

**Figure 1:**
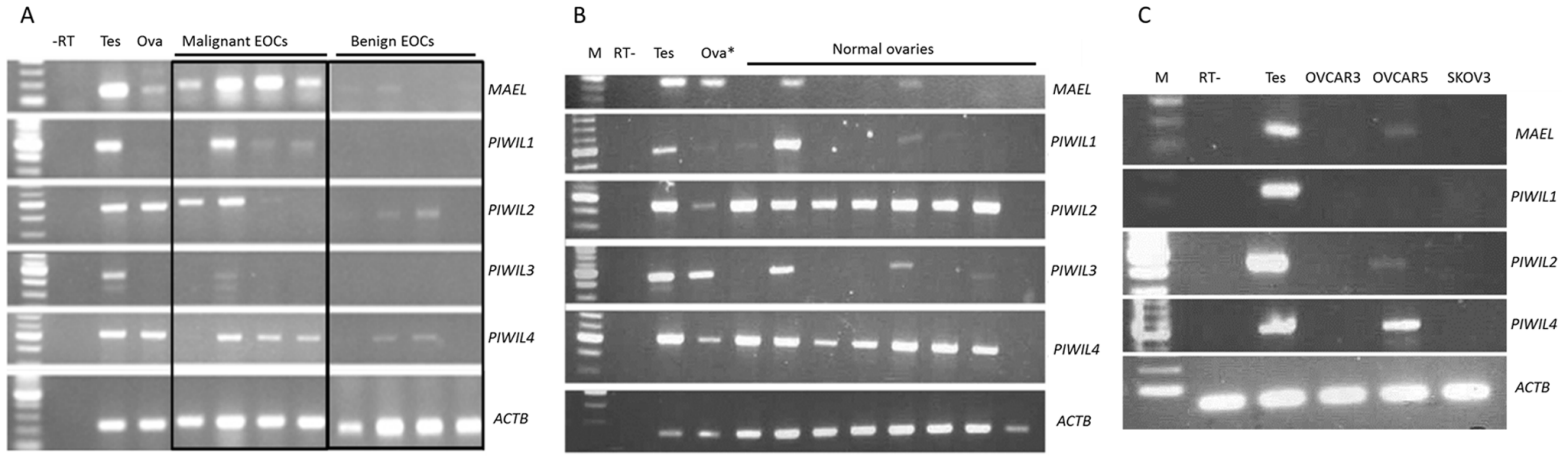
piRNA pathway gene expression in human normal ovaries and ovarian cancers. RT-PCR analyses of *PIWIL1-4* and *MAEL* expression in (A) malignant EOC, benign ovarian cancers (B) normal ovaries and (C) ovarian cancer cell lines. This is a representation of 4 out of 25 malignant EOC and 19 benign ovarian cancer tissues. Increased expression of *PIWIL1, 2, 4* and *MAEL*, but not *PIWIL3*, are found in malignant cancers compared to benign tumors. All normal ovaries show *PIWIL2* and *PIWIL4* expression but only some have *PIWIL1, 3* and *MAEL* expression. No endogenous *PIWIL1, 2, 4* and *MAEL* expression is detected in OVCAR3 and SKOV3 cells. Ova* indicates tissue from a 20-year old pre-menopausal ovary while other ovaries are from individuals aged 44–76 years old.

The *MAEL* (CCDS1257) and *PIWIL1* (CCDS9268) cDNA sequences were obtained from the NCBI CCDS database. Full-length cDNA sequence was amplified using the Expand High Fidelity PCR system (Roche) according to the manufacturer's protocol and cloned into a pcDNA3.1 mammalian expression vector. Plasmid DNAs including pcDNA 3.1 (Invitrogen) or pEGFP-N1 empty vectors (Clontech) were transfected into SKOV3 cells using Lipofectamine LTX (Invitrogen) following the manufacturer's instruction. SKOV3 cells were selected due to their endogenous *L1* expression levels and the fact that they showed no piRNA pathway gene expression (Fig. 1C). For each transfection, 8×10^4^ SKOV3 cells were seeded onto a 24-well plate 14 hrs before transfection and were cultured in RPMI 1640/5% FBS without Penicillin-streptomycin. Plasmid DNA (500 ng) was diluted in 100µl of Opti-MEM medium (Invitrogen) and incubated with 0.5µl Plus reagent (Invitrogen) and 2µl Lipofectamine LTX (Invitrogen) for 30 mins before being added into each well. After 6 hrs, the cell culture medium was replaced with new RPMI 1640/5% FBS medium and cells were incubated for 24 hrs at 37°C, 5% CO_2_ before harvesting for *in vitro* invasion analyses. The transfection of pEGFP-N1 empty vectors into SKOV3 cells allowed the transfection efficiency to be measured, as successfully transfected cells expressed the GFP protein. The pEGFP-N1 vector was subjected to the same growth and transfection conditions as outlined above, to determine the transfection efficiency which was approximately 70% after 24 hrs.

### *In vitro* invasion analyses with xCelligence

Cell invasion was tested with real-time invasion assay monitoring using CIM devices and the xCelligence DP system (Roche Diagnostics) [28]. Briefly, 4 hrs before the invasion assay, a CIM plate (Roche) was coated with 1∶20 diluted Growth factor reduced Matrigel basement membrane matrix (∼450µg/ml) (BD Biosciences). Then 40,000 cells, untransfected or transfected with empty vector (Ev) or *MAEL* or *PIWIL1* vectors, were seeded into each coated well. Cell activity was followed over a time period of 72 hrs by measuring the impedance signal in the CIM plate. The cell activity was recorded every minute in the first 12 hrs and every 5 mins for the following 12 hrs. Then from 24 hrs onwards until the end of the experiment, cell activity was recorded every 30 mins. In each CIM plate, triplicates of each group were performed to obtain the mean and standard deviation. The experiment was repeated three times.

### *PIWIL1* transcript sequencing

The PIWI domain of *PIWIL1* transcript was PCR amplified and products of different size were cloned into pGEM-T Easy vector (Promega). A total of 30 positive colonies from different PCR bands were selected from each ligation reaction and plasmid DNAs were purified using standard alkaline lysis methods (Qiagen) [29]. 200 ng of plasmid DNA were sequenced (Big Dye Terminator v3.1 Cycle Sequencing Kit, Applied Biosystems) using SP6 and T7 universal primers.

## Results

### Expression of piRNA pathway genes is increased in malignant EOC compared to benign tumors or normal ovarian tissues

**Figure 2:**
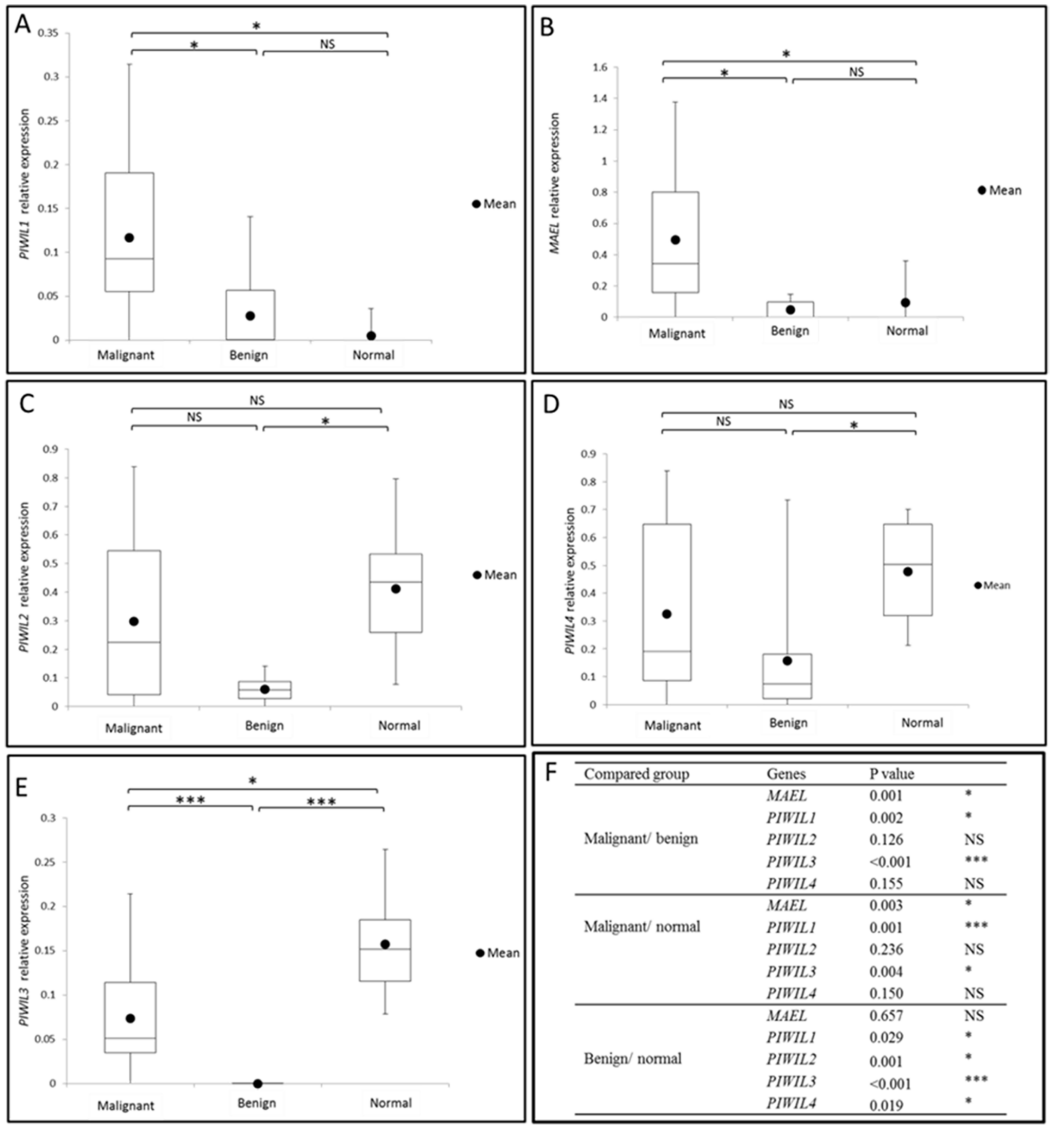
Box plot representing the expression of piRNA pathway genes in malignant EOC, benign ovarian cancer tissues and normal ovaries. Semi-quantitative RT-PCR analysis of mRNA expression (relative to *ACTB*) of (A) *PIWIL1*, (B) *MAEL*, (C) *PIWIL2*, (D) *PIWIL4* and (E) *PIWIL3*. (F) P-value of each comparison group. Malignant EOC (n = 25), benign ovarian cancer tissues (n = 19) and normal ovaries (n = 8). Kruskal-Wallis Test was performed to compare the gene expression level between two groups. Filled dot indicates mean of each group. * indicates 0.001<P<0.05; *** indicates P<0.001; NS, not significant.

piRNA pathway genes are consistently expressed in the mammalian ovary [11]. In order to investigate a possible role of this pathway in EOC, we performed semi-quantitative RT-PCR to investigate the expression of *PIWIL1*, *PIWIL2*, *PIWIL3*, *PIWIL4* and *MAEL* in advanced stage serous EOC (n = 25), benign ovarian tumors (n = 19) and normal ovarian tissue (n = 8) (Table 1, Table S3 and Fig. 1A, B). Semi quantitative analysis revealed that the relative expression levels of *PIWIL1* and *MAEL* were significantly higher in malignant EOC compared to benign and normal tissues (Fig. 2A, B). The relative expression levels of *PIWIL2* and *PIWIL4* in malignant groups were not significantly different to that in benign or normal ovarian tissues (Fig. 2C, D). However, the expression of *PIWIL2* and *PIWIL4* are significantly lower in benign tumors compared to normal ovarian tissue, suggesting an alteration of gene expression during the progression of EOC. The relative expression of *PIWIL3* is different to other *PIWIL* genes such that the expression of this gene is significantly higher in the normal ovary when compared to both malignant and benign tissues (Fig. 2E). Furthermore, the relative expression of *PIWIL3* in normal ovary is positively correlated with patient age in the normal ovary tissues (n = 8) (r = 0.750, P = 0.05) (Table 2). In contrast to *PIWIL3*, *PIWIL1* expression is negatively correlated with the age of patients (r = −0.737, P = 0.037). *PIWIL1* is expressed in growing follicles in pre-menopausal ovaries, but in this study half of the ovaries tested were from women less than 50 years old and likely to be premenopausal.

**Table 2:** Correlation between age of patients and piRNA pathway gene expression.

Genes	R-value (M, n = 25)	P-value (M, n = 25)	R-value (B, n = 19)	P-value (B, n = 19)	R-value (N, n = 8)	P-value (B, n = 19)
*PIWIL1*	−0.376	0.064	−0.194	0.426	−0.737	0.037*
*PIWIL2*	−0.080	0.702	−0.442	0.058	0.491	0.217
*PIWIL3*	−0.082	0.695	0.595	0.009*	0.75	0.05
*PIWIL4*	−0.249	0.231	−0.376	0.113	0	1.0
*MAEL*	−0.087	0.678	0.251	0.457	−0.220	0.601

Spearman's correlation test; M  =  malignant; B  =  benign; N  =  normal; negative value  =  negative correlation; positive value  =  positive correlation; P <0.05 and * indicates the correlation between the age of patients and piRNA pathway gene expression is significant. The R-value closer to ± 1 indicates the stronger correlation.

### *L1* and piRNA pathway genes are over expressed in EOC cells

**Figure 3:**
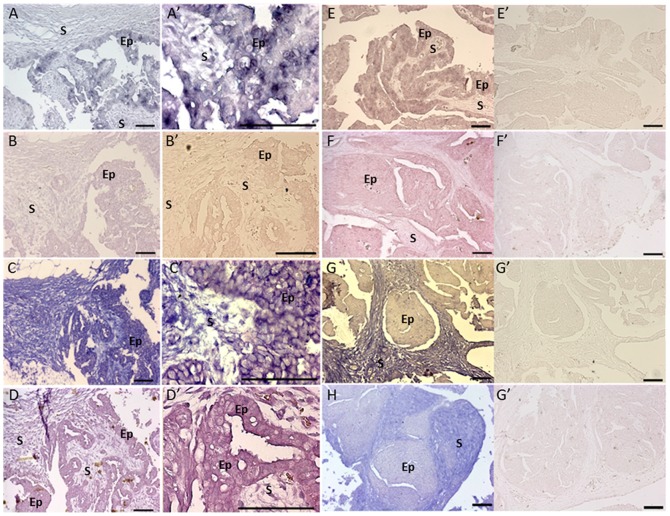
*L1*, *PIWIL1*, *MAEL* and *PIWIL2* are expressed in the epithelial cells of malignant EOC. *In situ* analysis of (A, A’, E) *L1*, (B, B’, F) *PIWIL1*, (C, C’, G) *MAEL*, (D, D’, H) *PIWIL2* in two malignant EOC (A-D from SC3 while E-H from SC2). (A’-D’) Amplification images of A-D respectively. Strong expression of piRNA pathway genes and *L1* was found in the squamous-to-cuboidal like epithelial cells of both malignant EOC except *PIWIL1* which only expressed in SC2 but not SC3. *L1* has patchy expression in some EOC such that some epithelial cells seem to have stronger *L1* expression compared to others. *MAEL* and *PIWIL2* but not *PIWIL1* are also expressed in the stromal cells in both EOC. (E’-H’) Negative controls with sense probe of *L1*, *PIWIL1*, *MAEL* and *PIWIL2* respectively. Ep  =  epithelial cells; S  =  stromal cells. Scale bar  =  50µm.

To analyse the expression pattern of piRNA pathway genes and *L1* in EOC, RNA *in situ* hybridization was performed in malignant EOC (n = 5) and benign ovarian tumors (n = 2) (Table 3, Fig. S1). We found strong expression of *L1* in the epithelial cells but *L1* was absent in the stromal cells in all samples (Fig. 3A, E). Also we noted that the expression of *L1* is variable in different patients (Fig. 3A). Strong expression of *PIWIL1* was found in the epithelial cells in all EOC tissues (Fig. 3B, F) while *MAEL* and *PIWIL2* showed strong expression in the epithelial cells and stromal cells of all EOC examined (Fig. 3C, G and D, H respectively).

**Table 3:** Expression trends of piRNA pathway genes and *L1* in the EOC after *in situ* hybridisation.

Patient	Gene	Epithelial cells	Stromal cells
SC1	*MAEL*	weak	strong
	*PIWIL2*	weak	strong
	*PIWIL1*	strong	-
	*L1*	strong	-
SC2	*MAEL*	weak	strong
	*PIWIL2*	weak	strong
	*PIWIL1*	weak	-
	*L1*	strong	-
SC3	*MAEL*	strong	strong
	*PIWIL2*	strong	strong
	*PIWIL1*	-	-
	*L1*	strong	weak
SC4	*MAEL*	-	-
	*PIWIL2*	strong	-
	*PIWIL1*	strong	-
	*L1*	strong	-
SC5	*MAEL*	weak	weak
	*PIWIL2*	weak	-
	*PIWIL1*	weak	-
	*L1*	weak	-
BSC1	*MAEL*	weak	weak
	*PIWIL2*	weak	weak
	*PIWIL1*	-	-
	*L1*	-	-
BSC2	*MAEL*	weak	-
	*PIWIL2*	weak	-
	*PIWIL1*	-	-
	*L1*	-	-

“-“: undetectable expression.

### Multiple *PIWIL1* transcript variants exist in malignant EOC

**Figure 4:**
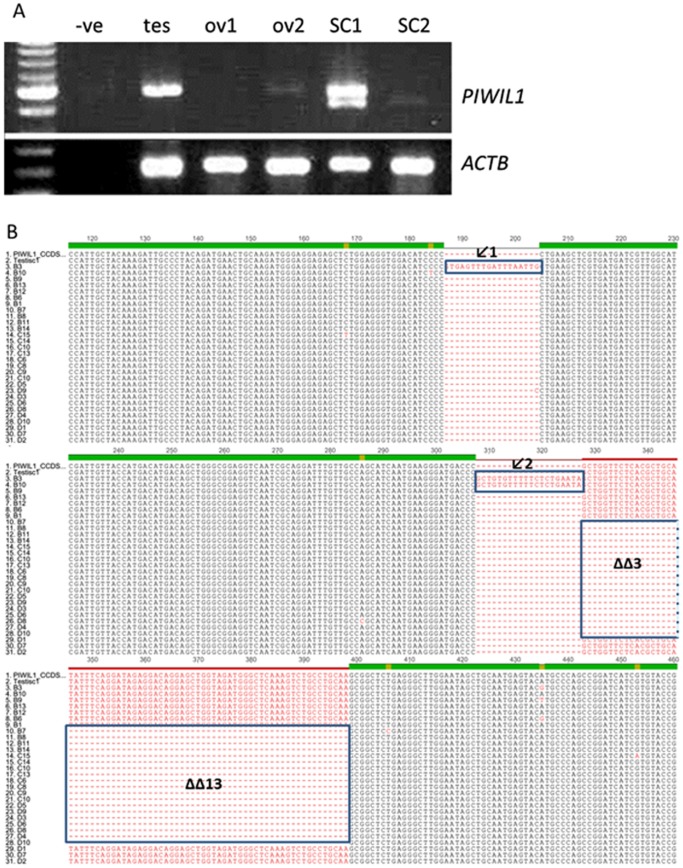
*PIWIL1* transcript variants in EOC. (A) *PIWIL1* expression in control tissues (human testis, normal ovary (ov) 1, 2) and EOC tissue SC1 and 2. In the positive control, a 500 bp band was amplified, but in malignant SC1, two bands were obtained. (B) cDNA multiple alignment (partial) showing that most of the clones have a deletion of exon 17 (ΔΔ3) and 3 clones have partial splicing of intron 15 and 16 (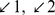). PIWIL1_ccds: published cDNA of *PIWIL1* (CCDS9268); Testis C1: testis transcript clone 1; B1–3, B6, B9–B14, C6, C8, C9, C10–C15, D1–D10: clones with *PIWIL1* transcripts.

PCR amplification of the PIWI domain from EOC cDNAs produced two distinct bands compared to cDNAs from normal testis or ovary (Fig. 4), suggesting the presence of multiple *PIWIL1* transcript variants in malignant EOC. PCR products from testis and malignant EOC were extracted, cloned and sequenced. Multiple alignments of clone sequences showed many changes within the PIWI domain at the nucleotide level when compared to the testis and published *PIWIL1* cDNA sequence. Single base pair changes have been identified in *PIWIL1* transcripts of malignant EOCs (Table S2). In addition, most of these clones have exon deletion and unspliced introns which introduce premature stop codons. Importantly a 75-nt deletion which comprised the entire exon 17 was found in most of the sequenced clones in 5 out of 28 EOC patients (Fig. 4B). Furthermore, some clones showed partial splicing of introns 15 and 16 (Fig. 4B) such that 17 bp and 20 bp from the 5′ end of intron 15 and 16, respectively, are unspliced. In order to confirm that the mutations are due to splicing, genomic DNA of tumor samples were isolated and sequenced at the corresponding region. No mutations were identified between exons 15–18 (where the splicing errors occurred) in the genomic *PIWIL1* sequence (Fig. S2). In order to predict if the above post-transcriptional alterations affect PIWIL1 function, sequences were translated and compared to the control (testis cDNA) and published peptide sequence (AAC97371.2). Loss of the entire exon 17 (73-nt deletion) in 11 clones introduced a premature stop codon in the PIWI domain (Fig. S3). Similarly, stop codons were found in clones that have unspliced introns (not shown).

### Over expression of piRNA pathway genes reduces invasiveness *in vitro*

**Figure 5:**
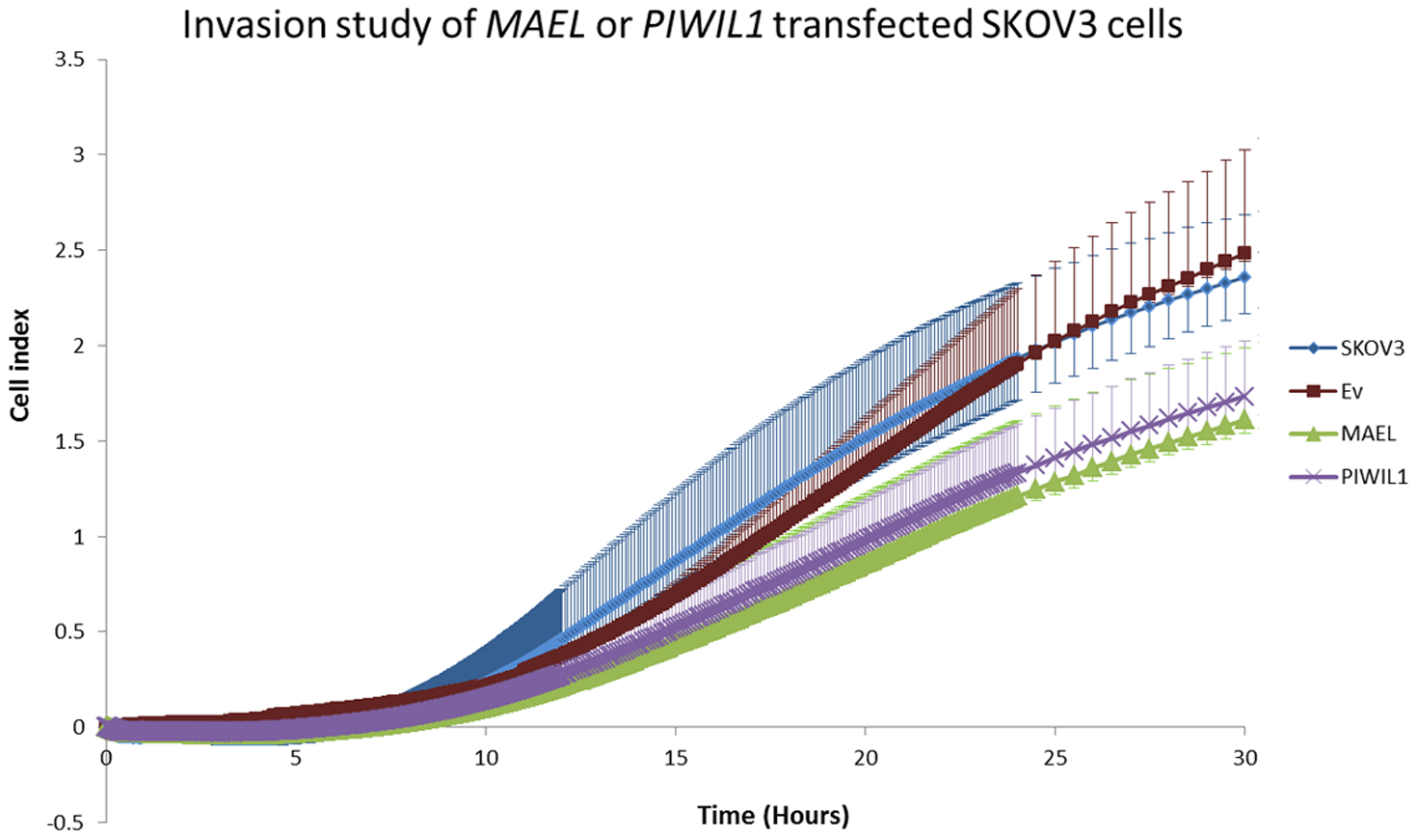
Invasion assay of *MAEL* or *PIWIL1* transfected SKOV3 cells. *PIWIL1* and *MAEL* transfected cells have lower invasiveness compared to untransfected cells and *Ev* transfected cells. In each experiment, each group was performed in triplicate and this experiment was repeated three times. This is a combined data plot of three independent experiments for all groups. Error bars represent standard deviation. *WT* indicates untransfected SKOV3 cells; empty vector (Ev), *MAEL* and *PIWIL1* indicate cells transfected with *MAEL* or *PIWIL1* vectors. *None* indicates well without cells.

In order to investigate the possible role of piRNA pathway genes in cancer progression, full-length *PIWIL1* or *MAEL* transcripts were cloned and transfected transiently into the ovarian cancer cell line SKOV3 and assayed for invasiveness after 24 hrs of transfection. RT-PCR was performed to validate the insertion of plasmids into these cells. RT-PCR confirmed *GFP*, *PIWIL1* or *MAEL* over expression (Fig. S4A). Expression of *MAEL* and *PIWIL1* constructs remained high in the transfected cells 50 hr post-transfection (Fig. S4A). *MAEL*, *PIWIL1* and empty vector (Ev) transfected cells and untransfected SKOV3 cells were applied to the xCELLigence system to investigate their effect on cell invasiveness [30]. Surprisingly, the invasiveness of *MAEL* and *PIWIL1* transfected cells was lower compared to untransfected or empty vector cells (Fig. 5). Cell invasiveness from each group started to reach a plateau after 30 hrs, thus only cell activity of the first 30 hrs is shown. Our results suggest that overexpression of *PIWIL1* and *MAEL* have a repressive effect on SKOV3 cell invasiveness.

## Discussion

The piRNA pathway is important for TE silencing, epigenetic regulation and stem cell self-renewal in a wide range of organisms [31], [32]. Global DNA hypomethylation and TE derepression, such as *L1,* is a common feature of cancer genomes [33], [34] in humans *PIWIL1* and *2* overexpression has been observed in tumors from various tissues [15]–[18], [21]–[23]. However, it is still unclear whether piRNA pathway genes are differentially expressed in ovarian cancer or have a role in ovarian tumor progression. We examined the expression of piRNA pathway genes *PIWIL1*–*4*, *MAEL* and *L1* in malignant EOC, benign tumors and normal ovary tissues, and also investigated possible roles of *PIWIL1* and *MAEL* in ovarian cancer cell lines.

Expression of *PIWIL1*–*2* has been investigated in various cancerous tissues [15]–[18], [21], [23], [35], but not *MAEL*, *PIWIL3* and *PIWIL4*. Although the expression of *PIWIL2* and *4* appeared high in tumor samples this was not significant, likely due to the fact that *PIWIL2* and *PIWIL4* were strongly expressed in normal ovarian tissues (Fig. 1B) and their expression among malignant tissues is highly variable. The expression of *PIWIL1* and *MAEL* however is significantly increased in malignant EOC when compared to benign tumors. *PIWIL1* and *MAEL,* but not *PIWIL2* and *4,* were significantly up regulated when compared to normal ovaries. In contrast to *PIWIL2* and *4* that are expressed in all normal ovarian tissues examined, the expression of *PIWIL1* and *MAEL* is found only in a subset of normal ovarian tissues from patients who were less than 50 years old. In normal ovary, *PIWIL1* and *MAEL* are expressed in the cumulus cells of growing follicles in human [11]. The normal ovarian tissues tested are from individuals aged 44–76 years old, and half of these samples were derived from individuals less than 50 years old. Thus, the variable expression of *PIWIL1* and *MAEL* in the normal ovaries could be explained by the difference in abundance of growing follicles, which express piRNA pathway genes across the samples.

*MAEL* is known as a cancer/testis gene as it is expressed in testis and a number of cancer cell lines [36]. Here we identified for the first time the increased expression of *MAEL* in malignant EOC and benign ovarian tumors. Similar to PIWI, MAEL is essential to ensure proper germline stem cell differentiation in *Drosophila* and other vertebrates [37]. Overexpression of piRNA pathway genes in EOC may indicate that some stem cell characteristics are present in tumor cells or that the piRNA pathway has been activated for example by activity of retrotransposons. Expression of these genes may also be a signature of the presence of ovarian cancer stem cells [38].

The expression of piRNA pathway genes in normal ovary and certain types of EOC may provide a new perspective on the origin of ovarian cancer. Somatic cells of the maturing follicle may come in contact with the ovarian surface epithelium during ovulation or may be present in the inclusion cyst which is thought to play a role in the origin of epithelial ovarian cancer [3] although the presence of inclusion cysts seems to not per se increase the risk of developing ovarian cancer [39]. Hypomethylation of the *L1* promoter region is correlated with increased *L1* mRNA expression, in malignant breast cancer tissues and cell lines [40], [41]. The expression of *PIWIL* genes and *MAEL* in both normal ovary and malignant EOC correlate with increased expression of repeat elements and raises the possibility that the piRNA pathway may have been triggered by TE expression. However, we noticed that *L1* expression was absent in earlier stage malignant tumors (n = 6) but consistently present at all stage 3c tumor samples. This provides some circumstantial evidence that the activation of piRNA pathway genes may precede *L1* expression during tumor progression. This is consistent with work in other tumors that correlate *L1* expression with cancer progression [42]. *Maelstrom* knock out results in decreased DNA methylation in gonads, but not in somatic tissue. In gonads this leads to a dramatic increase in transposable element expression in the germ cells [43]. Our observation of *MAEL* overexpression in the absence of detectable *L1* expression in early stage tumor samples raises the possibility that non-functional *MAEL* may play a role in decreasing DNA methylation promoting subsequent *L1* expression.

Although *PIWIL1* transcript level was significantly increased in malignant EOC compared to benign and normal tissues, cloning and sequencing of these transcripts suggested that these transcripts may produce aberrant and non-functional PIWIL1 protein. Multiple mutations were found in *PIWIL1* transcripts including unspliced introns, loss of exons as well as single base pair changes. Single base pair changes maybe a result of RNA editing [44]. Among thirty clones of *PIWIL1* transcripts from malignant tissues, one third of these clones have A to G (I) substitution. In addition, most of these clones have exon deletion and unspliced introns which introduced premature stop codons (Table 2). The presence of premature stop codons suggests that although *PIWIL1* expression is high in malignant tissues, the corresponding protein (if any) maybe truncated and non-functional. Such defects in pre-mRNA splicing have been found in liver cancer and have been implicated in its progression [45].

*In situ* analyses revealed that *L1*, *PIWIL1*, *PIWIL2* and *MAEL* are strongly expressed in the epithelial cells. Surprisingly we also found evidence for *PIWIL2* and *MAEL* expression in the stromal cells in malignant EOC. It is unclear at this point if these are epithelial cells invading stromal tissue or individual stromal cells expressing these genes. Several lines of evidence have demonstrated that alteration of gene expression in stromal cells may create a microenvironment which facilitates tumor growth, playing a role in cancer progression [46]–[48]. For example, changes of gene expression in the stromal cells surrounding colon cancer cells have been shown to produce matrix metalloproteinases (MMPs) which increase tumor invasion capacity *in vitro* and *in vivo* [47], [49].

There are a number of ways in which genes associated with the piRNA pathway can play a role in genome regulation and it has been suggested that activity of these genes can have effects on methylation and expression of TEs as well as genome instability. In addition, PIWI proteins can mediate, activating or repressing effects on chromatin via interaction with heterochromatin protein 1 or polycomb group proteins. Despite this experimental evidence elucidating the role of *PIWIL1* and *MAEL* in cancer is difficult as differing effects are observed. Recent studies suggest that *PIWIL1* maybe a marker for cancer cell proliferation as it is co-expressed with KI67 [16], a reliable proliferating cell marker [50]. In addition, studies in a *Drosophila* brain tumor model suggested that inactivation of *Piwi* suppressed tumor growth and thus *Piwi* may promote cancer progression in this system [51]. This is further supported by slowed tumor growth after knockdown of *PIWIL1* in lung cancer cell lines [52]. Recently several other studies investigated the effect of *PIWI* and *MAEL* overexpression on tumor progression. Liu et al observed increased expression of the *Maelstrom* gene in hepatocellular cancer (HCC). When overexpressed, *MAEL* led to increased growth, migration and invasiveness in HCC cell lines [53]. Liang et al. showed in another study, that stable overexpression of *Piwil2* in mouse embryonic fibroblasts increased proliferation and invasiveness [54]. However, in humans over expression of *PIWIL1* did not increase cell growth but caused programmed cell death in myeloid leukaemia KG1 cells, suggesting that PIWIL1 may prevent tumor development in these cells [55]. Our results in SKOV3 cells support those findings. Together this suggests that MAEL may undergo different roles depending on cell type and differentiation status. Given that these results were obtained in different cell types and tested in different assays and species it is difficult to reconcile the divergent results. One possible pathway that may shed some light on this is the AKT-GSK3b-Snail pathway. Recent work showed that *MAEL* overexpression triggers EMT via the AKT-GSK3b-SNAIL pathway. It has been suggested that activation of AKT will lead to degradation of SNAIL ultimately leading to EMT and increased invasiveness. However in SKOV3 cells *SNAIL* overexpression has been reported to increase invasiveness [56]. The decrease of invasiveness after *MAEL* overexpression observed in the present study could be due to a reduction in SNAIL activity by MAEL.

## Conclusions

Increasing evidence of piRNA pathway activity in various cancers raises questions about a role of this pathway in the origin and progression of these malignancies. As reported in this study, over expression of piRNA pathway genes and *L1* elements in malignant ovarian cancer suggest a role of this pathway in EOC. Expression of *PIWIL1* and *MAEL* is significantly up regulated in malignant EOC when compared to benign lesions and normal ovaries. *In situ* analyses revealed that *L1*, *PIWIL1*, *PIWIL2* and *MAEL* are strongly expressed in the cancerous cells but surprisingly *MAEL* and *PIWIL2* expression was also found in the stromal cells lining tumor tissues, suggesting a change in cell composition or identity in the tissue surrounding the cancer cells. Identification of aberrant *PIWIL1* transcript revealed that non-functional PIWIL1 proteins may be produced. In contrast to other cell systems, *in vitro* real-time invasion assay showed that over expression of piRNA pathway components such as *PIWIL1* and *MAEL* has a repressive effect on ovarian cancer cell invasiveness. Together, these results highlight the complexity in which the piRNA pathway may influence tumor progression and warrant further work to better understand the role of the piRNA pathway in the origin and progression of ovarian cancer.

## Supporting Information

Figure S1 **Expression of piRNA pathway genes and** ***L1*** **in malignant EOC.** (A) *MAEL* antisense from SC2 which shows strong expression in stromal cells (+++) compared to epithelial cells which have weak expression. (B) *L1* expression in SC3. Epithelial cells have patchy strong expression of *L1* while low expression was observed in the stromal cells. +++ strong expression; + weak expression. (A’–B’) Negative controls with a sense probe of *MAEL* and *L1* respectively. Scale bar = 50µm
(TIF)

Figure S2 **No mutations found in the genomic** ***PIWIL1*** **sequence in serous carcinoma 1.** SC1 gDNA was aligned with published *PIWIL1* gDNA (ENSG00000125207) sequence from exon 15 to exon 18. All exon sequences and partial intron sequences were shown. Green bar indicates 100% conservation between the aligned sequences.
(TIF)

Figure S3 ***PIWIL1*** **transcript variants encoded premature stop codon.** (A) 19 clones with exon 17 (ΔΔ13) deletions, 3 clones with unspliced introns and 1 clone with a single base change resulted in the introduction of premature stop codons. PIWIL1_ccds9268: published PIWIL1 peptide sequence (AAC97371.2); Testis C1: translated testis clone 1; B1-3, B6, B9-B14, C6, C8, C9, C10-C15, D1-D10: clones with PIWIL1 translated sequence. * inside the boxes in panel A indicates premature stop codon.
(TIF)

Figure S4 **Expression of** ***PIWIL1*** **and** ***MAEL*** **is maintained in transfected cells throughout the invasion study.** (A) RT-PCR showing the expression of *GFP*, *MAEL* and *PIWIL1* in transfected cells (left panel) at the start of invasion study (24 hrs post-transfection) and (right panel) the end of invasion study (74 hrs after transfection). The expression of *GFP, MAEL* or *PIWIL1* can only be detected in specific vector transfected cells but not empty vector transfected or wildtype cells. (B) A high number of GFP positive cells were still observed after 74 hrs of transfection. Scale bar = 10µm.
(TIF)

Table S1 **Primers for RT-PCR and *in situ* hybridization (ISH).**
(DOCX)

Table S2 **Nucleotide changes in PIWI domain of *PIWIL1* transcripts.**
(DOCX)

Table S3 **Classification of the individual tumor samples used.**
(DOCX)

## References

[1] Anttonen M, Ketola I, Parviainen H, Pusa AK, Heikinheimo M (2003) FOG-2 and GATA-4 Are coexpressed in the mouse ovary and can modulate mullerian-inhibiting substance expression. Biol Reprod 68: 1333–1340. 12606418 10.1095/biolreprod.102.008599

[2] Jemal A, Siegel R, Ward E, Hao Y, Xu J, et al (2008) Cancer statistics, 2008. CA Cancer J Clin 58: 71–96. 18287387 10.3322/CA.2007.0010

[3] Ricciardelli C, Oehler MK (2009) Diverse molecular pathways in ovarian cancer and their clinical significance. Maturitas 62: 270–275. 19193504 10.1016/j.maturitas.2009.01.001

[4] Feinberg AP, Vogelstein B (1983) Hypomethylation distinguishes genes of some human cancers from their normal counterparts. Nature 301: 89–92. 6185846 10.1038/301089a0

[5] Lander ES, Linton LM, Birren B, Nusbaum C, Zody MC, et al (2001) Initial sequencing and analysis of the human genome. Nature 409: 860–921. 11237011 10.1038/35057062

[6] Cordaux R, Batzer MA (2009) The impact of retrotransposons on human genome evolution. Nature reviews Genetics 10: 691–703. 10.1038/nrg2640 PMC2884099 19763152

[7] Smallwood SA, Kelsey G (2012) De novo DNA methylation: a germ cell perspective. Trends Genet 28: 33–42. 22019337 10.1016/j.tig.2011.09.004

[8] Aravin AA, Bourc'his D (2008) Small RNA guides for de novo DNA methylation in mammalian germ cells. Genes & development 22: 970–975. 18413711 10.1101/gad.1669408 PMC2732394

[9] Sarot E, Payen-Groschene G, Bucheton A, Pelisson A (2004) Evidence for a piwi-dependent RNA silencing of the gypsy endogenous retrovirus by the Drosophila melanogaster flamenco gene. Genetics 166: 1313–1321. 15082550 10.1534/genetics.166.3.1313 PMC1470774

[10] Deng W, Lin H (2002) miwi, a murine homolog of piwi, encodes a cytoplasmic protein essential for spermatogenesis. Dev Cell 2: 819–830. 12062093 10.1016/s1534-5807(02)00165-x

[11] Lim SL, Tsend-Ayush E, Kortschak RD, Jacob R, Ricciardelli C, et al (2013) Conservation and Expression of piRNA Pathway Genes in Male and Female Adult Gonad of Amniotes. Biol Reprod. 10.1095/biolreprod.113.111211 24108303

[12] Kuramochi-Miyagawa S, Watanabe T, Gotoh K, Totoki Y, Toyoda A, et al (2008) DNA methylation of retrotransposon genes is regulated by Piwi family members MILI and MIWI2 in murine fetal testes. Genes Dev 22: 908–917. 18381894 10.1101/gad.1640708 PMC2279202

[13] Zhang D, Xiong H, Shan J, Xia X, Trudeau VL (2008) Functional insight into Maelstrom in the germline piRNA pathway: a unique domain homologous to the DnaQ-H 3′-5′ exonuclease, its lineage-specific expansion/loss and evolutionarily active site switch. Biol Direct 3: 48. 19032786 10.1186/1745-6150-3-48 PMC2628886

[14] Aravin AA, Sachidanandam R, Girard A, Fejes-Toth K, Hannon GJ (2007) Developmentally regulated piRNA clusters implicate MILI in transposon control. Science 316: 744–747. 17446352 10.1126/science.1142612

[15] Qiao D, Zeeman AM, Deng W, Looijenga LH, Lin H (2002) Molecular characterization of hiwi, a human member of the piwi gene family whose overexpression is correlated to seminomas. Oncogene 21: 3988–3999. 12037681 10.1038/sj.onc.1205505

[16] Liu X, Sun Y, Guo J, Ma H, Li J, et al (2006) Expression of hiwi gene in human gastric cancer was associated with proliferation of cancer cells. International journal of cancer Journal international du cancer 118: 1922–1929. 16287078 10.1002/ijc.21575

[17] Liu JJ, Shen R, Chen L, Ye Y, He G, et al (2010) Piwil2 is expressed in various stages of breast cancers and has the potential to be used as a novel biomarker. International journal of clinical and experimental pathology 3: 328–337. 20490325 PMC2872741

[18] Lee JH, Engel W, Nayernia K (2006) Stem cell protein Piwil2 modulates expression of murine spermatogonial stem cell expressed genes. Molecular reproduction and development 73: 173–179. 16261612 10.1002/mrd.20391

[19] Chen C, Liu J, Xu G (2013) Overexpression of PIWI proteins in human stage III epithelial ovarian cancer with lymph node metastasis. Cancer Biomark 13: 315–321. 24440970 10.3233/CBM-130360 PMC12928306

[20] Janic A, Mendizabal L, Llamazares S, Rossell D, Gonzalez C (2010) Ectopic expression of germline genes drives malignant brain tumor growth in Drosophila. Science 330: 1824–1827. 21205669 10.1126/science.1195481

[21] Sun G, Wang Y, Sun L, Luo H, Liu N, et al (2011) Clinical significance of Hiwi gene expression in gliomas. Brain research 1373: 183–188. 21138738 10.1016/j.brainres.2010.11.097

[22] Grochola LF, Greither T, Taubert H, Moller P, Knippschild U, et al (2008) The stem cell-associated Hiwi gene in human adenocarcinoma of the pancreas: expression and risk of tumour-related death. British journal of cancer 99: 1083–1088. 18781170 10.1038/sj.bjc.6604653 PMC2567072

[23] Taubert H, Greither T, Kaushal D, Wurl P, Bache M, et al (2007) Expression of the stem cell self-renewal gene Hiwi and risk of tumour-related death in patients with soft-tissue sarcoma. Oncogene 26: 1098–1100. 16953229 10.1038/sj.onc.1209880

[24] Li L, Yu C, Gao H, Li Y (2010) Argonaute proteins: potential biomarkers for human colon cancer. BMC cancer 10: 38. 20146808 10.1186/1471-2407-10-38 PMC2843668

[25] Xiao L, Wang Y, Zhou Y, Sun Y, Sun W, et al (2010) Identification of a novel human cancer/testis gene MAEL that is regulated by DNA methylation. Mol Biol Rep 37: 2355–2360. 19693694 10.1007/s11033-009-9741-x

[26] Shuangshoti S, Hourpai N, Pumsuk U, Mutirangura A (2007) Line-1 hypomethylation in multistage carcinogenesis of the uterine cervix. Asian Pacific journal of cancer prevention: APJCP 8: 307–309. 17696752

[27] Iramaneerat K, Rattanatunyong P, Khemapech N, Triratanachat S, Mutirangura A (2011) HERV-K hypomethylation in ovarian clear cell carcinoma is associated with a poor prognosis and platinum resistance. International journal of gynecological cancer: official journal of the International Gynecological Cancer Society 21: 51–57. 21330831 10.1097/IGC.0b013e3182021c1a

[28] Xi B, Yu N, Wang X, Xu X, Abassi YA (2008) The application of cell-based label-free technology in drug discovery. Biotechnology journal 3: 484–495. 18412175 10.1002/biot.200800020

[29] Wolf CR, Hayward IP, Lawrie SS, Buckton K, McIntyre MA, et al (1987) Cellular heterogeneity and drug resistance in two ovarian adenocarcinoma cell lines derived from a single patient. Int J Cancer 39: 695–702. 3583449 10.1002/ijc.2910390607

[30] Eisenberg MC, Kim Y, Li R, Ackerman WE, Kniss DA, et al (2011) Mechanistic modeling of the effects of myoferlin on tumor cell invasion. Proceedings of the National Academy of Sciences of the United States of America. 10.1073/pnas.1116327108 PMC3250187 22135466

[31] Cox DN, Chao A, Baker J, Chang L, Qiao D, et al (1998) A novel class of evolutionarily conserved genes defined by piwi are essential for stem cell self-renewal. Genes & development 12: 3715–3727. 9851978 10.1101/gad.12.23.3715 PMC317255

[32] Siomi MC, Sato K, Pezic D, Aravin AA (2011) PIWI-interacting small RNAs: the vanguard of genome defence. Nature reviews Molecular cell biology 12: 246–258. 21427766 10.1038/nrm3089

[33] Roman-Gomez J, Jimenez-Velasco A, Agirre X, Cervantes F, Sanchez J, et al (2005) Promoter hypomethylation of the LINE-1 retrotransposable elements activates sense/antisense transcription and marks the progression of chronic myeloid leukemia. Oncogene 24: 7213–7223. 16170379 10.1038/sj.onc.1208866

[34] Piskareva O, Lackington W, Lemass D, Hendrick C, Doolan P, et al (2011) The human L1 element: a potential biomarker in cancer prognosis, current status and future directions. Current molecular medicine 11: 286–303. 21506922 10.2174/156652411795677954

[35] Grochola LF, Greither T, Taubert H, Moller P, Knippschild U, et al (2008) The stem cell-associated Hiwi gene in human adenocarcinoma of the pancreas: expression and risk of tumour-related death. Br J Cancer 99: 1083–1088. 18781170 10.1038/sj.bjc.6604653 PMC2567072

[36] Xiao L, Wang Y, Zhou Y, Sun Y, Sun W, et al (2010) Identification of a novel human cancer/testis gene MAEL that is regulated by DNA methylation. Molecular biology reports 37: 2355–2360. 19693694 10.1007/s11033-009-9741-x

[37] Pek JW, Lim AK, Kai T (2009) Drosophila maelstrom ensures proper germline stem cell lineage differentiation by repressing microRNA-7. Dev Cell 17: 417–424. 19758565 10.1016/j.devcel.2009.07.017

[38] Foster R, Buckanovich RJ, Rueda BR (2013) Ovarian cancer stem cells: working towards the root of stemness. Cancer Lett 338: 147–157. 23138176 10.1016/j.canlet.2012.10.023

[39] Sharma A, Apostolidou S, Burnell M, Campbell S, Habib M, et al (2012) Risk of epithelial ovarian cancer in asymptomatic women with ultrasound-detected ovarian masses: a prospective cohort study within the UK collaborative trial of ovarian cancer screening (UKCTOCS). Ultrasound Obstet Gynecol 40: 338–344. 22911637 10.1002/uog.12270

[40] Alves G, Tatro A, Fanning T (1996) Differential methylation of human LINE-1 retrotransposons in malignant cells. Gene 176: 39–44. 8918229 10.1016/0378-1119(96)00205-3

[41] Asch HL, Eliacin E, Fanning TG, Connolly JL, Bratthauer G, et al (1996) Comparative expression of the LINE-1 p40 protein in human breast carcinomas and normal breast tissues. Oncol Res 8: 239–247. 8895199

[42] Gualtieri A, Andreola F, Sciamanna I, Sinibaldi-Vallebona P, Serafino A, et al (2013) Increased expression and copy number amplification of LINE-1 and SINE B1 retrotransposable elements in murine mammary carcinoma progression. Oncotarget 4: 1882–1893. 24231191 10.18632/oncotarget.1188 PMC3875756

[43] Soper SF, van der Heijden GW, Hardiman TC, Goodheart M, Martin SL, et al (2008) Mouse maelstrom, a component of nuage, is essential for spermatogenesis and transposon repression in meiosis. Dev Cell 15: 285–297. 18694567 10.1016/j.devcel.2008.05.015 PMC2546488

[44] Bass BL (2002) RNA editing by adenosine deaminases that act on RNA. Annu Rev Biochem 71: 817–846. 12045112 10.1146/annurev.biochem.71.110601.135501 PMC1823043

[45] Berasain C, Goni S, Castillo J, Latasa MU, Prieto J, et al (2010) Impairment of pre-mRNA splicing in liver disease: mechanisms and consequences. World J Gastroenterol 16: 3091–3102. 20593494 10.3748/wjg.v16.i25.3091 PMC2896746

[46] Stahtea XN, Roussidis AE, Kanakis I, Tzanakakis GN, Chalkiadakis G, et al (2007) Imatinib inhibits colorectal cancer cell growth and suppresses stromal-induced growth stimulation, MT1-MMP expression and pro-MMP2 activation. Int J Cancer 121: 2808–2814. 17721919 10.1002/ijc.23029

[47] Gilles C, Polette M, Piette J, Munaut C, Thompson EW, et al (1996) High level of MT-MMP expression is associated with invasiveness of cervical cancer cells. Int J Cancer 65: 209–213. 8567119 10.1002/(SICI)1097-0215(19960117)65:2<209::AID-IJC14>3.0.CO;2-8

[48] Cirri P, Chiarugi P (2011) Cancer associated fibroblasts: the dark side of the coin. Am J Cancer Res 1: 482–497. 21984967 PMC3186047

[49] Okada A, Bellocq JP, Rouyer N, Chenard MP, Rio MC, et al (1995) Membrane-type matrix metalloproteinase (MT-MMP) gene is expressed in stromal cells of human colon, breast, and head and neck carcinomas. Proc Natl Acad Sci U S A 92: 2730–2734. 7708715 10.1073/pnas.92.7.2730 PMC42292

[50] Brown DC, Gatter KC (2002) Ki67 protein: the immaculate deception? Histopathology 40: 2–11. 11903593 10.1046/j.1365-2559.2002.01343.x

[51] Janic A, Mendizabal L, Llamazares S, Rossell D, Gonzalez C (2010) Ectopic expression of germline genes drives malignant brain tumor growth in Drosophila. Science 330: 1824–1827. 21205669 10.1126/science.1195481

[52] Liang D, Dong M, Hu LJ, Fang ZH, Xu X, et al (2013) Hiwi knockdown inhibits the growth of lung cancer in nude mice. Asian Pac J Cancer Prev 14: 1067–1072. 23621188 10.7314/apjcp.2013.14.2.1067

[53] Liu L, Dai Y, Chen J, Zeng T, Li Y, et al (2014) Maelstrom promotes hepatocellular carcinoma metastasis by inducing epithelial-mesenchymal transition by way of Akt/GSK-3beta/Snail signaling. Hepatology 59: 531–543. 23929794 10.1002/hep.26677

[54] Shahali M, Kabir-Salmani M, Nayernia K, Soleimanpour-Lichaei HR, Vasei M, et al (2013) A novel in vitro model for cancer stem cell culture using ectopically expressed piwil2 stable cell line. Cell J 15: 250–257. 24027667 PMC3769608

[55] Sharma AK, Nelson MC, Brandt JE, Wessman M, Mahmud N, et al (2001) Human CD34(+) stem cells express the hiwi gene, a human homologue of the Drosophila gene piwi. Blood 97: 426–434. 11154219 10.1182/blood.v97.2.426

[56] Lu ZY, Dong R, Li D, Li WB, Xu FQ, et al (2012) SNAI1 overexpression induces stemness and promotes ovarian cancer cell invasion and metastasis. Oncol Rep 27: 1587–1591. 22344746 10.3892/or.2012.1685

