# Supplementary figures and images for: Overexpression of piRNA Pathway Genes in Epithelial Ovarian Cancer
Source: PLoS One. 2014 Jun 16;9(6):e99687. doi: 10.1371/journal.pone.0099687 (PMC4059699; doi:10.1371/journal.pone.0099687)

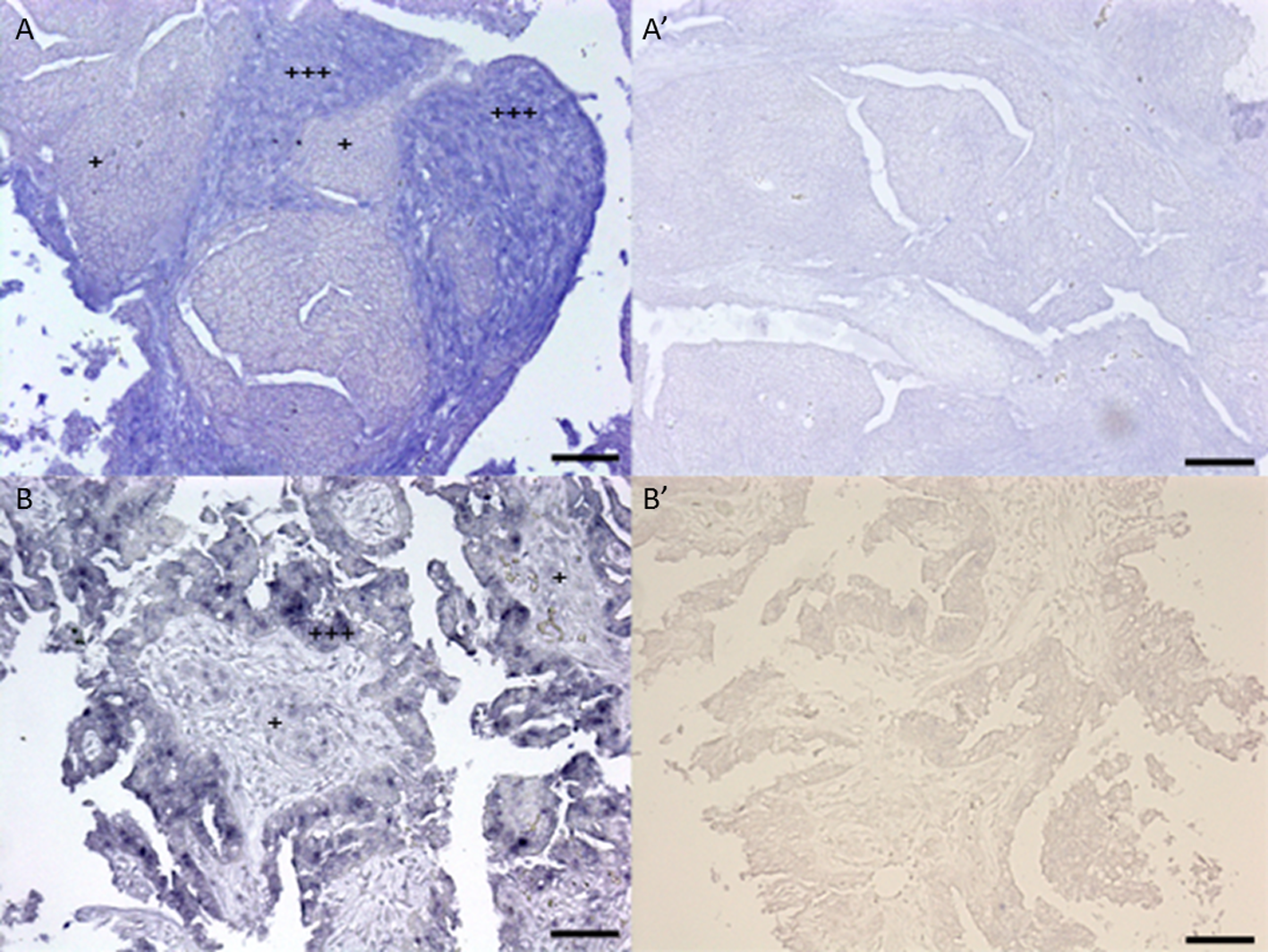

Supplement: Figure S1 — Expression of piRNA pathway genes and L1 in malignant EOC. (A) MAEL antisense from SC2 which shows strong expression in stromal cells (+++) compared to epithelial cells which have weak expression. (B) L1 expression in SC3. Epithelial cells have patchy strong expression of L1 while low expression was observed in the stromal cells. +++ strong expression; + weak expression. (A’–B’) Negative controls with a sense probe of MAEL and L1 respectively. Scale bar = 50µm (TIF) [file pone.0099687.s001.tif]

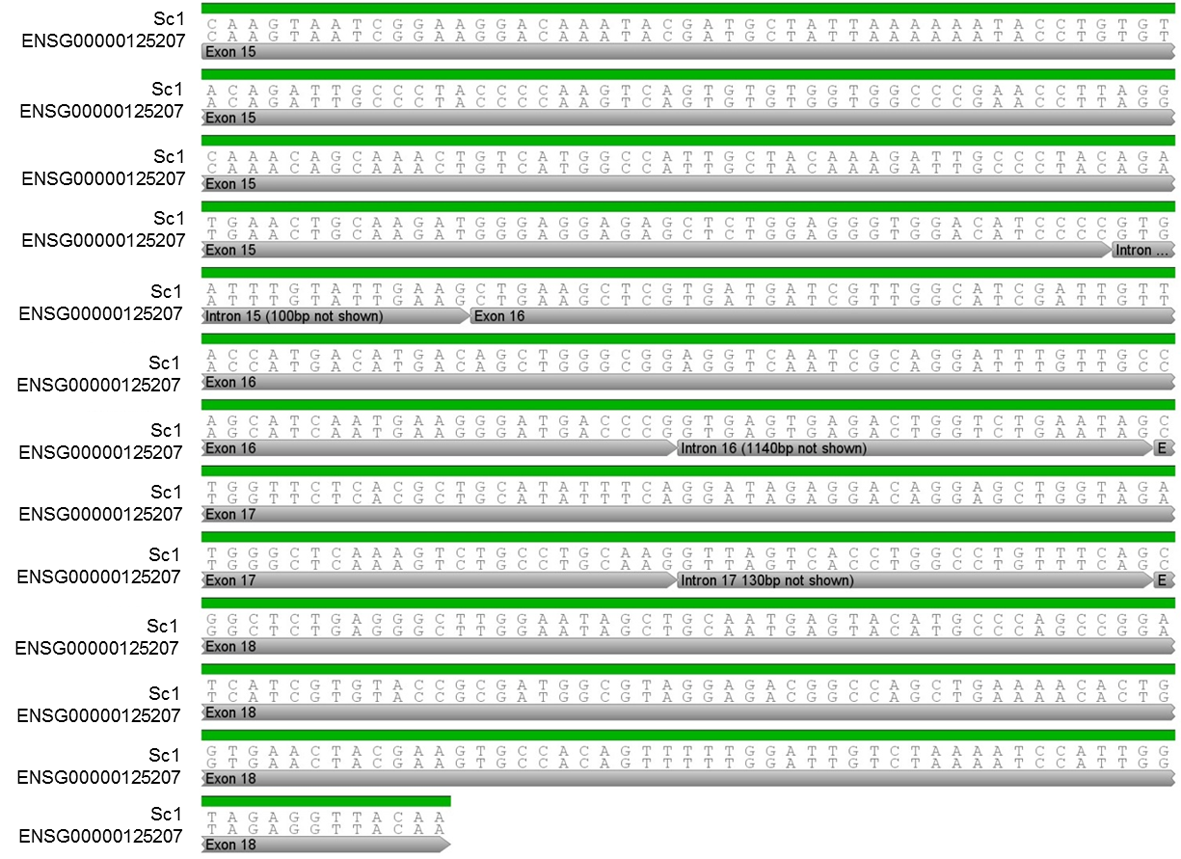

Supplement: Figure S2 — No mutations found in the genomic PIWIL1 sequence in serous carcinoma 1. SC1 gDNA was aligned with published PIWIL1 gDNA (ENSG00000125207) sequence from exon 15 to exon 18. All exon sequences and partial intron sequences were shown. Green bar indicates 100% conservation between the aligned sequences. (TIF) [file pone.0099687.s002.tif]

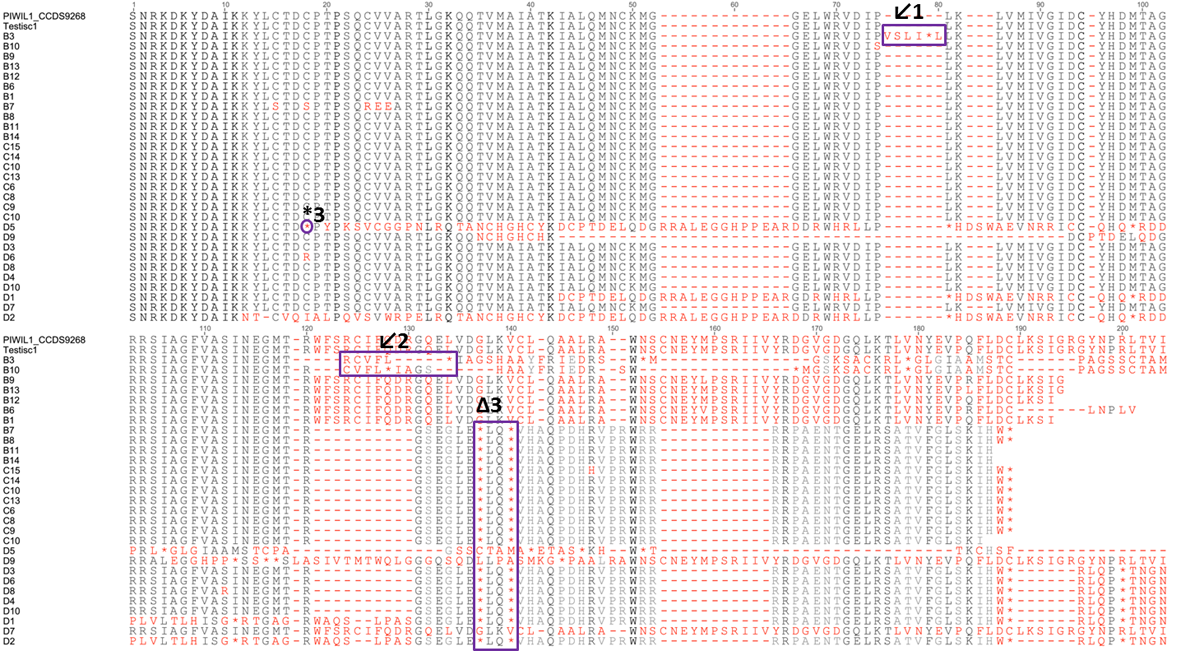

Supplement: Figure S3 — PIWIL1 transcript variants encoded premature stop codon. (A) 19 clones with exon 17 (ΔΔ13) deletions, 3 clones with unspliced introns and 1 clone with a single base change resulted in the introduction of premature stop codons. PIWIL1_ccds9268: published PIWIL1 peptide sequence (AAC97371.2); Testis C1: translated testis clone 1; B1-3, B6, B9-B14, C6, C8, C9, C10-C15, D1-D10: clones with PIWIL1 translated sequence. * inside the boxes in panel A indicates premature stop codon. (TIF) [file pone.0099687.s003.tif]

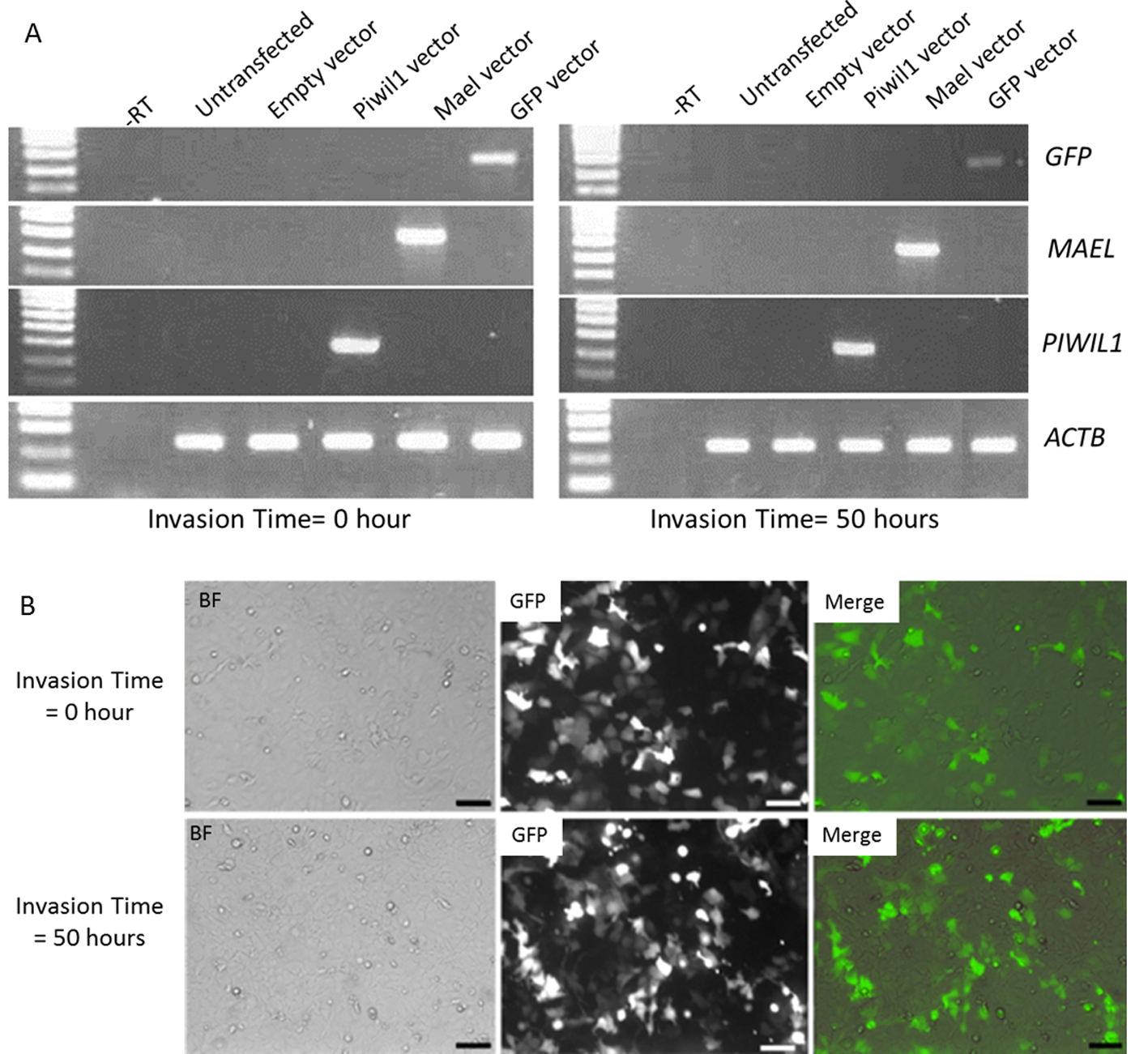

Supplement: Figure S4 — Expression of PIWIL1 and MAEL is maintained in transfected cells throughout the invasion study. (A) RT-PCR showing the expression of GFP, MAEL and PIWIL1 in transfected cells (left panel) at the start of invasion study (24 hrs post-transfection) and (right panel) the end of invasion study (74 hrs after transfection). The expression of GFP, MAEL or PIWIL1 can only be detected in specific vector transfected cells but not empty vector transfected or wildtype cells. (B) A high number of GFP positive cells were still observed after 74 hrs of transfection. Scale bar = 10µm. (TIF) [file pone.0099687.s004.tif]
