# Supplementary material for: Overexpression of piRNA Pathway Genes in Epithelial Ovarian Cancer
Source: PLoS One. 2014 Jun 16;9(6):e99687. doi: 10.1371/journal.pone.0099687 (PMC4059699; doi:10.1371/journal.pone.0099687)
Supplement: Table S1 — Primers for RT-PCR and in situ hybridization (ISH). (DOCX) [file pone.0099687.s005.docx]

Table S1: Primers for RT-PCR and *in situ* hybridization (ISH).

| Primer | Sequence 5’-3’ | Tm | Experiment |
| --- | --- | --- | --- |
| *MAEL* ex7 forward | CTGATGATAGAACCAGAGTC | 55˚C | ISH |
| *MAEL* ex8 reverse | GAATCCAAGTCTTAGAGGGC | 55˚C | RT-PCR |
| *MAEL* ex11 reverse | TAGAAGAGTTGAAATGAGAGAA | 55˚C | ISH, RT-PCR |
| *PIWIL1* ex15 forward | CAAGTAATCGGAAGGACAAA | 52˚C | ISH, RT-PCR |
| *PIWIL1* ex18 reverse | CTACCAATGGATTTTAGACAA | 52˚C | ISH, RT-PCR |
| *PIWIL2* ex3 forward | AACAGTTTCTAAGACCCCTC | 55˚C | ISH |
| *PIWIL2* ex5 forward | TACCTTCAGCACACCGTCC | 55˚C | RT-PCR |
| *PIWIL2* ex6 reverse | GACACTGTATTTTGACGAGGT | 55˚C | RT-PCR |
| *PIWIL3* ex15 forward | GGTGATTTGTATCCTGCCCA | 55˚C | RT-PCR |
| *PIWIL3* ex18 reverse | TGACCATCTCCCACTCCATC | 55˚C | RT-PCR |
| *PIWIL4* ex9 forward | TACTGTATCGGACCTGAATCAG | 55˚C | RT-PCR |
| *PIWIL4* ex10 reverse | TTCAGCCACAGCCTTCATCAG | 55˚C | RT-PCR |
| *ACTB* ex6 forward | GCCGTCTTCCCCTCCATCGT | 55˚C | RT-PCR |
| *ACTB* ex7 reverse | CCTCGGTCAGCAGCACGGGG | 55˚C | RT-PCR |
| *L1* forward | CTCAAAGGAAAGCCCATCAG | 52˚C | ISH, RT-PCR |
| *L1* reverse | CGTGAGATGGGTTTCCTGA | 52˚C | ISH, RT-PCR |
