# Supplementary material for: Overexpression of piRNA Pathway Genes in Epithelial Ovarian Cancer
Source: PLoS One. 2014 Jun 16;9(6):e99687. doi: 10.1371/journal.pone.0099687 (PMC4059699; doi:10.1371/journal.pone.0099687)
Supplement: Table S2 — Nucleotide changes in PIWI domain of PIWIL1 transcripts. (DOCX) [file pone.0099687.s006.docx]

Table S2: Nucleotide changes in PIWI domain of *PIWIL1* transcripts.

| Mutation loci (bp from the start of the PIWI domain) | RNA editing | Clones |
| --- | --- | --- |
| 9 loci (69, 532, 555, 849, 877, 889, 910, 921, 923) | A→G | B3, B6, B7, B9, B10, B13, D3, D6, D8, D9 |
| 6 loci (186, 202, 452, 499, 509, 529) | C→T | B3, B6, B9, B13, C15, |
| 4 loci (64, 73, 530, 887) | A→T | B7, B9, B10, D9 |
| 5 loci (91, 93, 95, 98, 952) | T→A | B7, D8 |
| 3 loci (109, 849, 897) | T→C | C14, D2, D7 |
