# Supplementary material for: Overexpression of piRNA Pathway Genes in Epithelial Ovarian Cancer
Source: PLoS One. 2014 Jun 16;9(6):e99687. doi: 10.1371/journal.pone.0099687 (PMC4059699; doi:10.1371/journal.pone.0099687)
Supplement: Table S3 — Classification of the individual tumor samples used. (DOCX) [file pone.0099687.s007.docx]

Table S3: Classification of the individual tumor samples used.

| Samples | Cancer stage | Age | Tumour grade | Localisation | Primary or Metastasize |
| --- | --- | --- | --- | --- | --- |
| 1 | 3c | 61 | 3 | Ovary | Primary |
| 2 | 3c | 61 | 3 | Ovary | Primary |
| 3 | 3c | 50 | 3 | Ovary | Primary |
| 4 | 3c | 75 | 3 | Ovary | Primary |
| 5 | 3 | 46 | 3 | Ovary | Primary |
| 6 | 3c | 51 | 1 | Ovary | Primary |
| 7 | 1a | 54 | 2 | Ovary | Primary |
| 8 | 1a | 67 | 3 | Ovary | Primary |
| 9 | 1a | 37 | 1 | Ovary | Primary |
| 10 | 3c | 64 | 3 | Ovary | Primary |
| 11 | 2b | 73 | 3 | Ovary | Primary |
| 12 | 3c | 44 | 3 | Ovary | Primary |
| 13 | 1c | 58 | 3 | Ovary | Primary |
| 14 | 1c | 75 | 3 | Ovary | Primary |
| 15 | 3c | 59 | 3 | Ovary | Primary |
| 16 | 3c | 47 | 3 | Ovary | Primary |
| 17 | 3c | 63 | 3 | Ovary | Primary |
| 18 | 3c | 66 | 3 | Ovary | Primary |
| 19 | 3c | 76 | 3 | Ovary | Primary |
| 20 | 3c | 73 | 3 | Ovary | Primary |
| 21 | 3c | 74 | 3 | Tubal | Primary |
| 22 | 3c | 69 | 3 | Peritoneal | Primary |
| 23 | 3c | 69 | 3 | Ovary | Primary |
| 24 | 3b | 37 | 3 | Ovary | Primary |
| 25 | 3c | 60 | 3 | Ovary | Primary |
